# Integrative analysis to identify oncogenic gene expression changes associated with copy number variations of enhancer in ovarian cancer

**DOI:** 10.18632/oncotarget.21227

**Published:** 2017-09-23

**Authors:** Xiaoyan Li, Yining Liu, Jiachun Lu, Min Zhao

**Affiliations:** ^1^ Beijing Anzhen Hospital, Capital Medical University, Beijing Institute of Heart, Lung & Blood Vessel Disease, Beijing, China; ^2^ The School of Public Health, Institute for Chemical Carcinogenesis, Guangzhou Medical University, Guangzhou, China; ^3^ The School of Public Health, The First Affiliated Hospital, Guangzhou Medical University, Guangzhou, China; ^4^ School of Engineering, Faculty of Science, Health, Education and Engineering, University of the Sunshine Coast, Queensland, Australia

**Keywords:** systems biology, cancer genomics, enhancer, copy number variation

## Abstract

Enhancers are short regulatory regions (50-1500 bp) of DNA that control the tissue-specific activation of gene expression by long distance interaction with targeting gene regions. Recently, genome-wide identification of enhancers in diverse tissues and cell lines was achieved using high-throughput sequencing. Enhancers have been associated with malfunctions in cancer development resulting from point mutations in regulatory regions. However, the potential impact of copy number variations (CNVs) on enhancer regions is unknown. To learn more about the relationship between enhancers and cancer, we integrated the CNVs data on enhancers and explored their targeting gene expression pattern in high-grade ovarian cancer. Using human enhancer-gene interaction data with 13,691 interaction pairs between 7,905 enhancers and 5,297 targeting genes, we found that the 2,910 copy number gain events of enhancer are significantly correlated with the up-regulation of targeting genes. We further identified that a number of highly mutated super-enhancers, with concordant gene expression change on their targeting genes. We also identified 18 targeting genes by super-enhancers with prognostic significance for ovarian cancer, such as the tumour suppressor *CDKN1B*. We are the first to report that abundant copy number variations on enhancers could change the expression of their targeting genes which would be valuable for the design of enhancer-based cancer treatment strategy.

## INTRODUCTION

To establish and maintain specific physiological states in different developmental stages and cell types, gene expression is highly regulated by thousands of transcription factors (TFs), cofactors and chromatin regulators [1]. For accurate gene regulation, the TFs and cofactors are often binding in specific genomic regions that include promoters and enhancers. Compared to the proximal promoters for gene regulation, enhancers are often physically located up to 1Mbp away from the target genes [2]. In addition, these gene-distal regulatory elements can be upstream or downstream from the transcription starting site and either in the forward or reverse strand. In general, enhancers have more tissue specificity compared to promoter-based gene regulation [2].

As the most lethal gynecologic malignancy in women, ovarian cancer (OVC) can be classified as low-grade (well-differentiated) or high-grade (poorly differentiated) [3]. Due to the absence of symptoms in an early stage, OVC is regarded as a ‘silent killer’ with an estimation of 15,500 deaths in the United States in 2012 [3]. Although the steady accumulation of genetic and pathogenesis studies in OVC, there is rare focus on the interplay of regulatory events at promoter regions [4]. The roles of enhancer in ovarian cancers have been reported in only one recent publication and at single gene level [5].

Despite a number of studies on enhancer methylation dynamics in promoter regions of other cancer types [6], the global mutational patterns of enhancers are unknown. Due to the limitation of precise enhancer and target gene, the interaction of enhancers and their target genes in ovarian cancer are unexplored as well. Since recent studies confirm the dosage effect of copy number variation (CNV) in genes [7], we present the first systematic study of CNV in enhancer regions and explore the potential effects on their target genes. In summary, the integrative mutation and expression analyses was conducted in ovarian cancer to elucidate the relationship between enhancer CNVs and targeting gene expression changes in the hundreds of matched ovarian cancer samples from The Cancer Genome Atlas (TCGA) [8].

## RESULTS

### A computational framework to identify the gene expression change induced by copy number variation of enhancer

**Figure 1:**
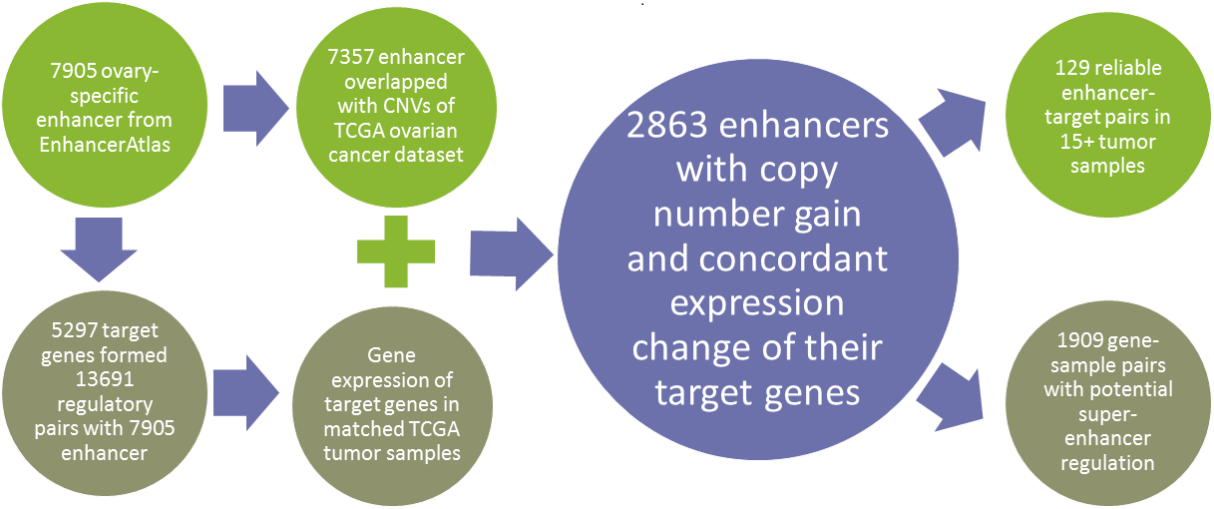
The computational pipeline to explore the CNV of enhancers

To explore the CNV of a specific DNA region, the genomic coordinate is necessary to intersect all the known CNV regions. Since enhancers are DNA fragment on the chromosome, they could easily to be represented as genomic coordinates. Based on the overlapping genomic coordinates, we mapped enhancers to CNV data. By further incorporating the gene expression of enhancer targeting genes, we could check whether the CNVs on enhancer have effects on the expression change of targeting genes. To survey the role of enhancers in ovarian cancer, we focused on the CNVs on enhancer regions and constructed a computational framework with extensive data integration (Figure 1, see Methods). We downloaded 7905 ovary-specific enhancers (Supplementary Table 1) from EnhancerAtlas that all human enhancers were predicted based on three or more independent high throughputs experimental evidence (e.g. histone modification, enhancer RNA, transcription factor binding, DNase I hypersensitive sites) [9]. In addition, EnhancerAtlas provided the predicted target genes. In order to use the reliable target genes, we set a limit of 0.7 for the confidence score and collected 5297 associated targeting genes with 7905 enhancers in ovary tissue (Supplementary Table 1). We then used genomic intersecting functionality from Bedtools to intersect each enhancer with those characterized CNVs from TCGA ovary cancer [10]. In total, we found 4620 CNVs associated with 7357 ovary enhancers in 507 TCGA ovary cancer samples (Supplementary Table 2). By checking the expression of those targeting genes in the same TCGA cancer samples, we identified those concordant regulatory pairs which reflect the CNV-based enhancer dosage effects on target gene expression. We also defined the super-enhancers if multiple enhancers in the chromosome targeting the same genes with up-regulating effects. By correlated those targeting genes to the human interaction network and the functional features of those targeting genes regulated by enhancers with copy number gain, we further associated key cancer signaling pathways with the enhancer CNV-based regulation. Finally, we surveyed those key genes with prognostic features associated with survival data and which provided a list of genes with potential clinical applications.

### The consistency of gene expression changes induced by CNVs in enhancer regions

There are 13,691 regulatory pairs with 7,905 ovary enhancers and 5,297 target genes according to enhancerAtlas (Supplementary Table 1). To evaluate the effect of the enhancers with CNVs, we integrated the gene expression data from the same TCGA ovarian cancer sample and evaluated the regulation of their associated genes. We further classified these CNV-based expression change based on CNV type and the concordance of the gene expression change. Since we aimed to explore the involvement of enhancer in targeting gene regulation, we only studied two possible concordant changes: enhancer copy number gain/gene up- regulation and copy number loss/gene down-regulation. We found 4,353 concordant regulatory pairs with copy number gain of enhancer and up-regulation of target genes in 212 TCGA tumor samples (Supplementary Table 3). By contrast, we found 1,037 pairs with copy number loss and consistent gene down-regulation in 61 TCGA tumor samples. In those enhancer-expression pairs, there were 2910 unique enhancers associated with gain/up-regulation and 887 associated with loss/down-regulation. Therefore, we further focused on the 4230 regulatory pairs with 2862 enhancer and 1743 target genes.

By counting the number of samples with the same concordant regulatory events, we identified 129 regulatory pairs detected in 15 or more ovary tumor samples (Supplementary Table 3). These 129 pairs consisted of 115 enhancers and 64 target genes. For example, the enhancer at “chr8:124034530-124034920” has the concordant regulatory effects on its target gene *DERL1* in 54 TCGA ovarian cancer samples. This target gene encoding Derlin-1 has been detected over-expressed in multiple cancers, including breast [11], colon [12], and non-small cell lung cancer [13]. Derlin-1 can participate in the ER-associated degradation response and retrotranslocate misfolded or unfolded proteins from the ER lumen to the cytosol for proteasomal degradation. This process may have clinical application as a novel cancer target in attempt to develop a new tumor targeting therapy [14]. These 64 genes are enriched in a number of cytobands (Supplementary Table 4) in 3q27, 8q24, 19p13 and 19q13, which may imply the potential active regions for enhancer regulation. These genes are also enriched in nuclear regions, such as nucleoplasm (corrected P-value = 0.00131), nuclear body (corrected P-value = 0.0285), and perinuclear region of cytoplasm (corrected P-value = 0.0429).

Usually, the gain of copy number in enhancer regions has multiple predicted targeting genes with concordant up-regulation in the matched ovarian cancer samples in TCGA. In our data, we identified two enhancers with seven target genes: “chr19:13189550-13190960” and “chr14:23316840-23319540” (Supplementary Table 1). For the enhancer in chromosome 19, the target genes are *TRMT1*, *TNPO2*, *NFIX*, *DNASE2*, *PRDX2*, *NANOS3*, and *LYL1*, which was detected in 22 unique tumor samples. Although the other one in chromosome 14 has 7 target genes, it was only detected in three unique tumor samples. In summary, the consistent expression changed in multiple tumor samples, potentially induced by enhancer, are ideal candidate therapeutic target that may be translated into clinical application.

### The identification of 210 target genes regulated by super-enhancers

A super-enhancer was defined as a cluster of enhancers located in a specific genomic region and that collectively drives transcription of genes [15]. To further examine the potential of super-enhancer regulation, we explored those genes targeted by multiple enhancers for each tumor sample. By using a cutoff of three proximal enhancers, we identified a total of 1909 gene-sample pairs with the potential to be subject to super-enhancer regulation (Supplementary Table 5). These regulations involved 585 target genes in 184 tumor samples. For example, the nuclear receptor corepressor 2 (*NCOR2*) has a cluster of 20 upstream enhancers detected in a sample (TCGA-24-1562-01) with amplifications. Although *NCOR2* was related to breast cancer [16], this regulation was only observed in a single tumor sample. To survey those reliable regulations with multiple samples-based evidence, we selected 210 target genes with an upstream super-enhancer in at least three tumor samples. Interestingly, the target genes are enriched in cytobands in chromosome 19 including 19p13.2 (21 genes, corrected P-value = 1.33E-16), 19p13.11 (8 genes, corrected P-value = 1.16E-6), 19p13.1 (6 genes, corrected P-value = 2.64E-5), 19q13.2 (8 genes, corrected P-value = 1.57E-4), and 19p13.1 (5 genes, corrected P-value = 1.57E-4). Cytoband 19p13.11 was associated with survival and susceptibility to ovarian cancer in a genome-wide study [17]. Additionally, the 210 genes are enriched in four gene families: Zinc fingers C2H2-type|PR/SET domain family (21 genes, corrected P-value = 2.79E-5); Pleckstrin homology domain containing (8 genes, corrected P-value = 1.29E-2); Proteasome (4 genes, corrected P-value = 1.29E-2); and MADS-box family (2 genes, corrected P-value = 1.60E-2). For the Zinc fingers C2H2-type|PR/SET domain family, one of its members, *ZHX1*, was regulated by a cluster of five enhancers in 22 samples, which covered 12% of 184 tumor sample with detected super-enhancer regulation. As a transcription repressor, *ZHX1* has been associated with the progressions of cholangiocarcinoma, hepatocellular carcinoma, gastric cancer, and breast cancer [18]. For Pleckstrin homology domain containing genes, there are two AKT proteins (*AKT1/2*), which regulate cell proliferation and growth. A mutation in *AKT1* has been implicated as cancer causation in breast, colorectal and ovarian cancers in humans [19].

We identified 142 over-represented pathways (Supplementary Table 6). The top enriched pathways including Epstein-Barr virus infection (corrected P-values = 1.61E-3), Prolactin Signaling Pathway (corrected P-values = 1.71E-3), G alpha 13 Pathway (corrected P-values = 1.71E-3), and G alpha q Pathway (corrected P-values = 5.22E-3). Some of the genes are shared in these top ranked pathways including *MYC*, *AKT1*, and *YWHAZ*. The gene *YWHAZ* is a member of the 14–3-3 gene family and participates in the epithelial-mesenchymal transition *via* PI3K-Akt signaling pathway by binding to phosphoserine-containing proteins [20]. It was regulated by a cluster of five enhancers in 16 tumor samples in our results (Supplementary Table 5).

**Figure 2:**
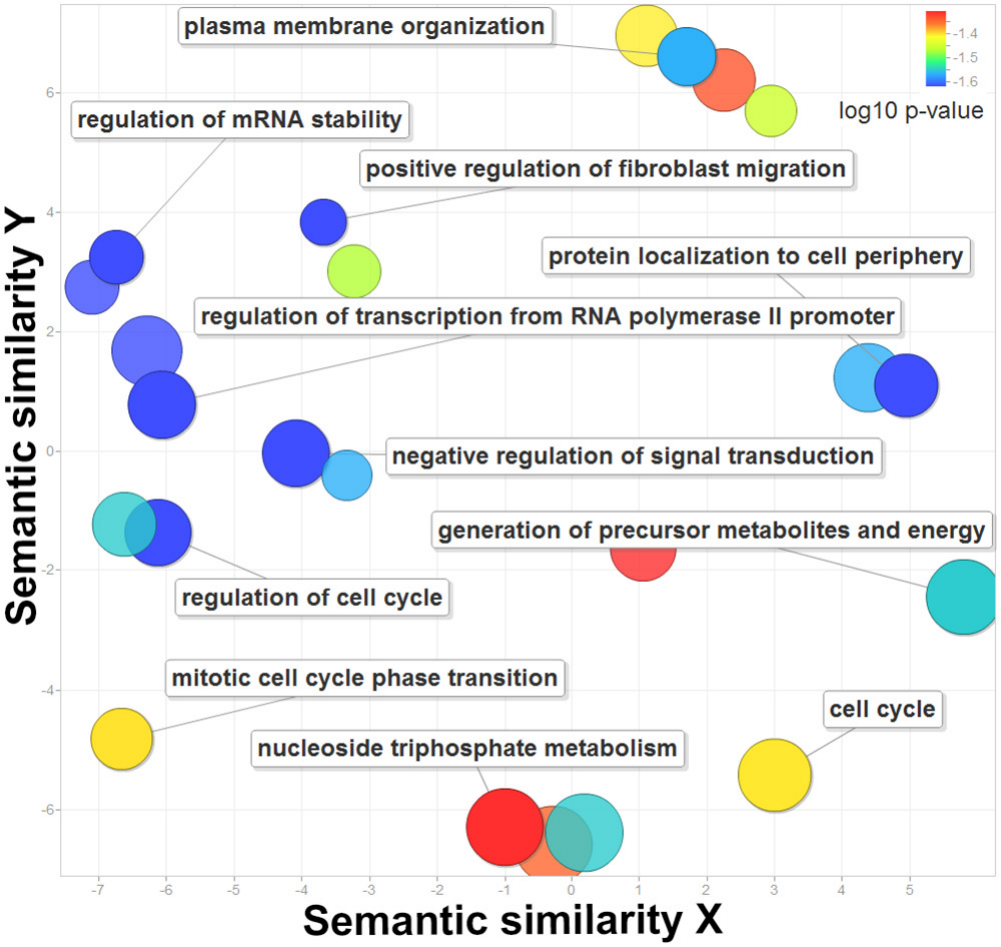
The enriched gene ontology terms for the 210 genes regulated by super-enhancers The X and Y axes represent the semantic similarities of the gene ontology terms. The log 10 of the corrected P-values are plotted in different colors.

From a gene ontology perspective, the 210 genes are associated with regulation of cell cycle, RNA stability and transcription from RNA polymerase II promoter (Figure 2, Supplementary Table 5, corrected P-values = 2.41E-2). They are also related to subcellular locations such as plasma membrane organization (corrected P-values = 2.73E-2) and protein localization to cell periphery (corrected P-values = 3.55E-2), which may help to identify multiple localizations with more functions [21]. In addition, these genes are rate-limiting enzymes in fundamental metabolism and energy production including generation of precursor metabolites and energy (corrected P-values = 2.84E-2) and nucleotide triphosphate metabolism (corrected P-values = 4.94E-2) [22]. In summary, the identified 210 genes regulated by super-enhancers play critical roles in basic gene regulation and metabolic and signaling pathways.

### The connected network of 210 target genes regulated by super-enhancers

**Figure 3:**
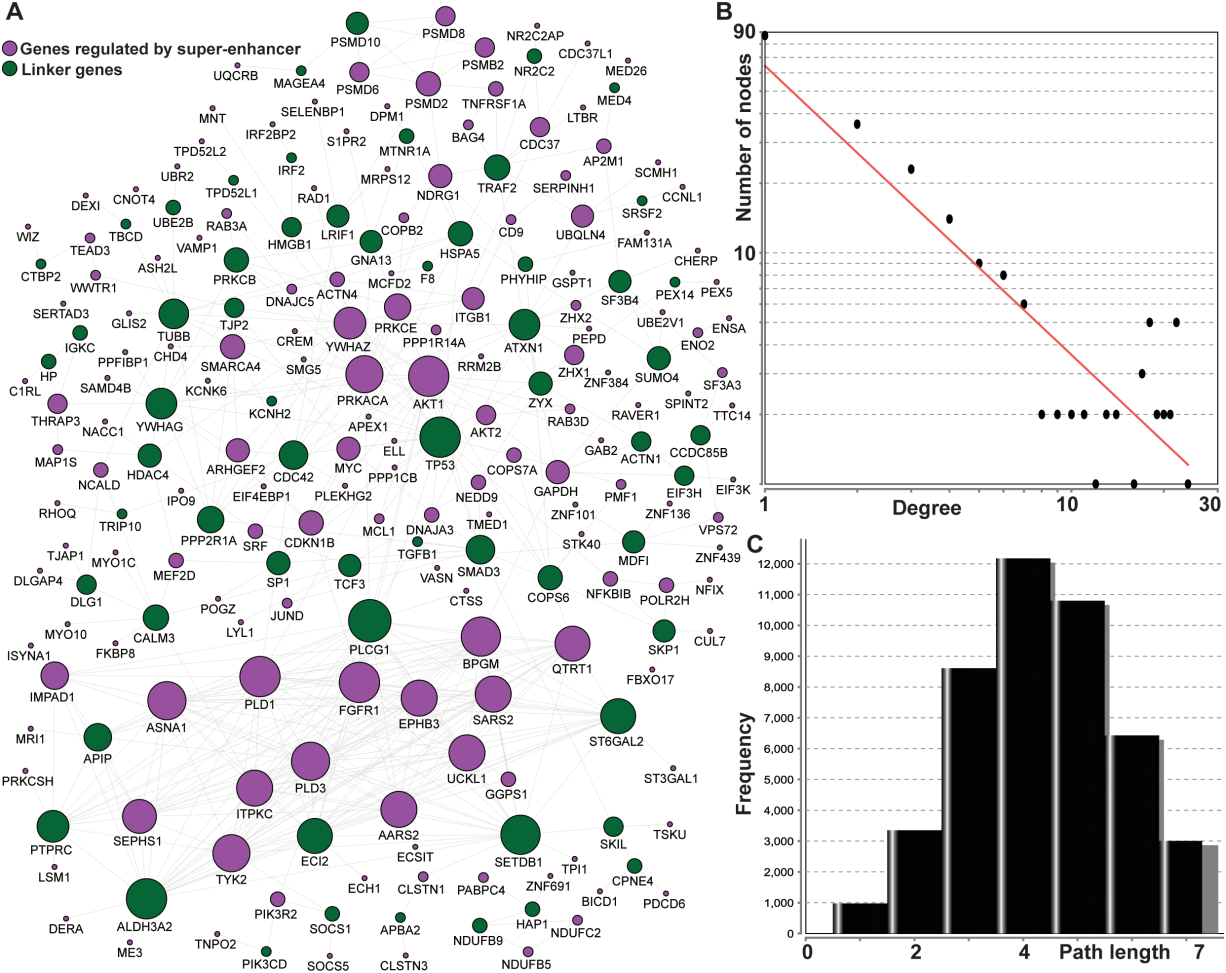
The interactome for 210 genes regulated by super-enhancers using pathway-based protein-protein interaction data **(A)** The 158 genes in blue are regulated by super-enhancers; the remaining 52 genes in green are linking genes to connect the 158 genes. The size of the node represents the number of connections in the network; **(B)** the degree distribution for the network; and **(C)** the short path length frequency for the network.

To further explore the potential common pathways associated with the 210 genes regulated by super-enhancers, we utilized the network approach to connect those genes. The Pathway Commons database was used to extract gene-gene functional interaction pairs [23]. The interactome from Pathway Commons was summarized from the knowledge of the cellular pathway for metabolism and signaling transduction in a few popular biological pathway databases such as KEGG [24] and Reactome [25]. Thus, the entire network was substantially smaller than that of the protein-protein interactome based on physical interactions, which have higher false-positives and are not useful for further pathway reconstruction. Based on the reliable human pathway-based interactome, we constructed a comprehensive cellular map for 210 genes regulated by super-enhancers. The reconstructed map contains 217 genes and 486 gene-gene interactions using evidence from known biological pathways (Figure 3A). One hundred and fifty-eight of the 217 nodes were from the 210 super-enhancer regulating genes. The remaining 59 are the linker genes that connect the 210 genes to fully express their cellular function. Approximately three-quarters of 210 genes are linked to each other in a highly modular structure and this suggests/indicates that the super-enhancers could regulate a highly-connecting cellular modular.

Further network topological analysis indicated that most molecules in our map were not sparsely connected. In general, the network diameter is 12, which characterizes the shortest distance between the two most distant nodes. On average, each node has 4.497 neighbors although 87 nodes possessed a single connection (Figure 3B). This means that 130 nodes can readily communicate with each via short steps. The number of connections (degrees) of all genes regulated by super-enhancers follows a power law distribution *P(k)∼k*^*-b*^, where *P(k)* is the probability that a gene has connections with other *k* genes and *b* is an exponent with an estimated value of 1.25. This means that the super-enhancer regulatory map is different from all other human interaction networks in which most of the genes are sparsely connected with exponent *b* as 2.9 [26]. This feature skewed the shortest path distribution for the whole network to a smaller number (3 - 5) and means that >53% of the short paths have three or fewer (Figure 3C). Next, we focused on the hub nodes in this network, which may be common connections facilitating mediate information transduction in thousands of short paths. We found eight genes, *PLCG1* (24), *AKT1* (22), *FGFR1* (22), *TP53* (22), *PLD1* (22), *ALDH3A2* (22), *SETDB1* (21), and *BPGM* (21) with over 20 connections. Four of these (*PLCG1* [27], *TP53*, *ALDH3A2* [28], *SETDB1* [29]) are linking genes which are not regulated by super-enhancers but are related to cancers. In summary, our network analysis of super-enhancer regulating genes discovered a highly connected functional module and provides links to critical cancer driver genes such as *PLCG1* and *TP53*. This relationship also highlights the important regulatory functions of super-enhancers.

### The prognostic features of 210 regulated by super-enhancers

**Figure 4:**
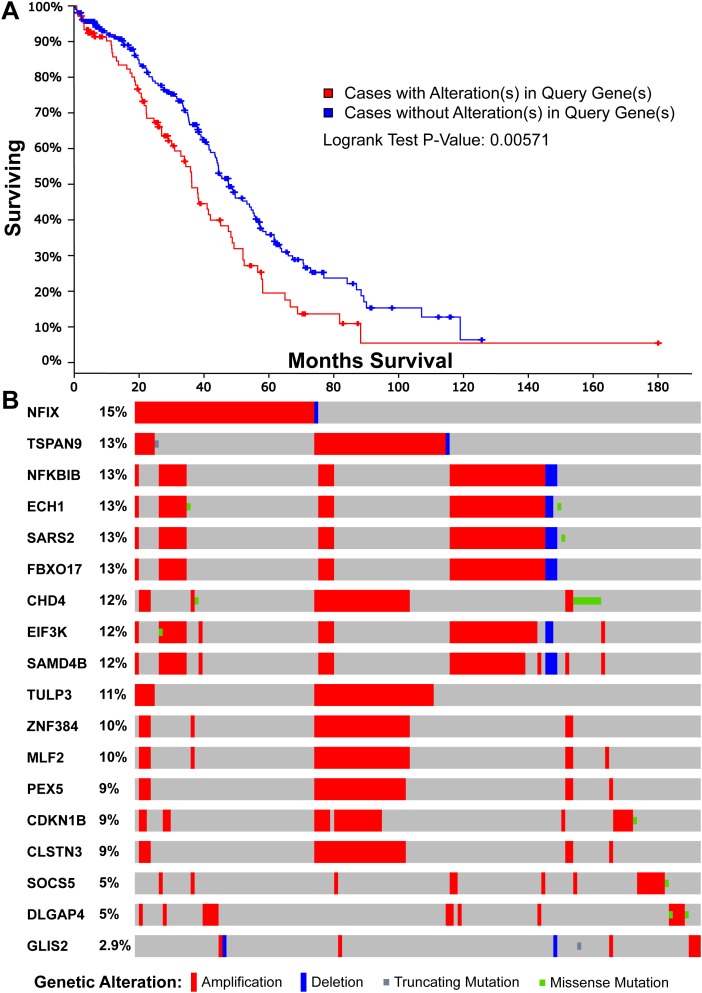
Survival and mutational analyses for the 18 genes with a significant prognostic feature in the TCGA ovarian cancer dataset **(A)** The overall survival characteristics of the 18 genes on the genetic mutation using cBio data portal [44]. **(B)** The sample-based oncoprint for the 18 genes in TCGA ovarian cancer dataset.

To explore the potential prognostic application of up-regulated target genes, we mapped these genes with the human prognostic database PRECOG which details survival outcomes [30]. For each cancer dataset, a Z-score was calculated to indicate the change of gene expression and associated clinical outcomes. By comparing Z-scores across multiple cancer datasets, we evaluated the prognostic potential for those target genes associated with super-enhancers (Supplementary Table 7). We found that 18 of the 210 genes gave a prognostic Z-score >1.96 in the TCGA ovarian cancer dataset; this is equivalent to a two-tailed P-value < 0.05. For example, *CDKN1B* is one of the 18 genes, which is known for its tumor suppressive role to regulate cell cycle [31] and stimulate regeneration [31]. However, it was also identified as an oncogene to cause stem cell expansion [32]. By performing the survival analysis using the mutation data from TCGA, we found that the survival time of patients with mutations in these 18 genes is significantly different from those without mutations (Figure 4A, P-value = 0.00571 for overall survival analysis).

Interestingly, the 18 genes with high prognostic value are enriched in a number of pathways. In particular, there are seven genes (*CDKN1B*, *PEX5*, *EIF3K*, *MLF2*, *NFKBIB*, *TULP3*, and *FBXO17*) associated with “Vitamin E” from drug association database (Corrected P-value = 4.73E-02) [33]. A previous study revealed that Vitamin E could suppress telomerase activity in ovarian cancer cells [34]. More interestingly, seven different genes (*ECH1*, *PEX5*, *MLF2*, *NFIX*, *TSPAN9*, *SAMD4B*, and *NFKBIB*) were associated with another drug C646, which is a small molecule inhibitor targeting histone acetyltransferase p300, an enzyme that alters the cancer transcriptome in the lung [35], colon and pancreas [36]. In relation to the mutational pattern in ovarian cancer, we plotted the sample-based mutations for all the 18 genes in TCGA ovarian cancer dataset (Figure 4B). The 18 genes are altered in 142 (46%) of 311 sequenced tumor samples with copy number variation data. Most of the mutations are amplifications, which suggests that these genes have potential oncogenic functions in progression of ovarian cancers. In addition, some of these 18 genes are close to each other and four (*EIF3K*, *SAMD4B*, *SARS2*, *FBXO17*) are from the same cytoband 19q13.2 (Corrected P-value = 5.89E-05). The other two genes *ECH1* and *NFKBIB* are from 19q13.1 (Corrected P-value = 3.99E-03). In summary, our analysis provides the insight into the potential prognostic significance of enhancers in ovarian cancer and the association with vitamin E and C646 may provide novel therapeutic ways to reduce tumorigenicity.

## DISCUSSION

Our data integration of human enhancers and targets provides the first comprehensive DNA-protein interactome in ovarian cancer. By overlapping this information with CNVs, we revealed several important somatic mutational features of enhancers, particularly with respect to the copy number gain. The integration of expression data from the matched TCGA samples, allows us to propose that potential mechanisms concerning the changes in enhancers’ copy number may be critical factors in inducing gene expression. A number of super-enhancers were identified and the prognostic analysis of corresponding targeting genes is the first report of a correlation between the neuropeptide system and clinical survival. The amplification of enhancers may be used for biomarkers in cancer and maybe a potential target for anti-cancer drug designing.

Due to the non-coding properties of enhancers, it was difficult to evaluate the functional impact of point mutations. Some of these enhancers may express as long non-coding RNA, which could be evaluate their functions using co-expressed genes [37]. Therefore, region-based mutations, such as CNV, were the basis of our study. The data quality of CNV may affect our results and interpretation. Firstly, some low-frequency CNVs may not be identified due to the small sample size of TCGA data (hundred individuals) [20]. Secondly, the CGH array-based CNV detection may not be able to characterize the outside regions of pre-designed probes, which may lose some signals in the enhancer regions. The further integration of large-scale CNV data and gene expression will provide new insights into the impacts of CNVs on enhancer-related regulation in cancers.

## MATERIALS AND METHODS

### The target genes of enhancers in human ovary tissue

To conduct a systematic CNV survey of ovarian cancer enhancers, we downloaded 7905 ovary-specific enhancers in a plain text format from EnhancerAtlas database [9]. The comprehensive database for human enhancers, EnhancerAtlas, contained enhancers’ annotation and analysis results in 105 human cell/tissue types. The cross-validation of enhancers was conducted for each cell/tissue type by integration of multiple experimental datasets with the relative weights (e.g. histone modification, enhancer RNA, transcription factor binding and DNase I hypersensitive sites). Therefore, we focused on the task to compare enhancers in a particular genomic region and assign enhancers and their target genes from the ovary-specific dataset.

To intersect enhancers with CNVs, we first extracted these 7905 ovary-specific enhancers with precise GRCH37 genomic locations. To correlate the CNV and the expression change, we downloaded targeting genes for all the 7905 enhancers. These potential targeting genes are predicted using a confidence score >0.7.

### The copy number variation and gene expression data from TCGA ovarian cancer cohort

To compile precise gain or loss of copy in enhancers, we downloaded the CNV data with the GRCH37 genomic coordinates from Catalogue of Somatic Mutations in Cancer database (COSMIC) (V78) [38]. In COSMIC, the copy number gain (CNG) was defined as either the average genome ploidy < = 2.7 AND total DNA segment copy number > = 5 or the average genome ploidy > 2.7 AND total DNA segment copy number > = 9. The comparable criteria for copy number loss (CNL) were as (the average genome ploidy < = 2.7 AND total DNA segment copy number = 0) OR (average genome ploidy > 2.7 AND total DNA segment copy number < (average genome ploidy −2.7)). An in-house shell script was implemented to extract the overlapping regions between ovary-specific enhancers and CNVs from the TCGA ovarian cancer samples. In total, we obtained 7,357 ovary enhancers with precise gain or loss information in 507 TCGA ovary cancer samples. To obtain reliable the gain or loss information, we use cross validation of the number of samples with CNG or CNL in the ovarian cancer samples. However, the majority of enhancers have substantial CNGs. Consequently, we collected enhancers with more than twice tumor samples with CNGs as those tumor samples with CNLs.

To investigate the targeting gene expression changes caused by enhancers, all the ovarian cancer gene expression profiles were downloaded from the COSMIC database (V78). To provide the accurate expression change analysis, we only used those gene expression data in matched TCGA samples with enhancer CNGs. The average and sample standard deviation for each gene were calculated based on the RSEM quantification results from the RNAseq V2 platforms. The standard Z-scores were applied to characterize whether a gene is over or under expressed in tumor samples. A Z-score of at least1.96 was used to define increased gene expression.

### The prognostic features of those up-regulated target genes

To explore the prognostic feature of enhancer targeting genes, the TCGA pan-cancer-based prognostic Z-scores were downloaded from PRECOG [30]. The data provided the prognostic relevance of the human protein coding genes by integrating gene expression, clinical survival data, and regulatory data. Across 39 cancer types, the Cox proportional hazards regressions were applied to each gene by focusing on gene-level expression and survival outcomes. Finally, standard Z-scores were used to determine whether the genes are associated with significant longer or shorter survival times in multiple cancers.

### The enhancer-related regulatory network construction and visualization

To explore the regulatory genes of super-enhancers in a pathway context, we extracted protein-protein interaction data for the 210 target genes regulated by super-enhancers. To provide the accurate interaction in biological pathways, a non-redundant pathway-based human interactome was built based on the known pathway-based interactions from HumanCyc [39], the NCI pathway interaction database [40], Reactome [25], and KEGG pathway database [24]. The final human pathway-based interactome contains 3,629 genes and 36,034 interacting edges, which is relatively small compared with the size of physical interactome without any biological implication. To construct a sub-network associated with the 210 super-enhancer targeting genes, we used the extraction approach described in our previous study [41]. This algorithm mapped all the 210 genes to the pathway-based interactome and then generated a sub-network with the shortest paths between 210 genes and other human genes.

Unlike the functional studies on a single gene, protein-protein interaction networks are often too complex to enable the function on each node to be assessed. However, because a small number of simple topological rules relate to network function, the topological properties of a network could be used to characterize its global function. For each gene in the network, we calculated the number of connections for a node, also known as the degree, and the short path which identifies the shortest steps for one gene to interact with another. All the topological analyses were conducted using the NetworkAnalyzer plugin in Cytoscape 2.8 [42] and the further network visualization and layout was performed using Cytoscape 2.8 as well. We systematically examined those genes in the network with regard to their functional gene ontology and visualized them using REVIGO [43]. The somatic mutational pattern in TCGA ovarian cancer cohort was visualized by using cBio portal [44].

## SUPPLEMENTARY MATERIALS TABLES

















## Abbreviations

CNV: Copy Number Variation
CNG: Copy Number Gain
CNL: Copy Number Loss
TCGA: The Cancer Genome Atlas
GO: Gene Ontology
ENCODE: Encyclopedia of DNA Elements
COSMIC: Catalogue Of Somatic Mutations In Cancer
