# Supplementary material for: Integrative analysis to identify oncogenic gene expression changes associated with copy number variations of enhancer in ovarian cancer
Source: Oncotarget. 2017 Sep 23;8(53):91558–67. doi: 10.18632/oncotarget.21227 (PMC5710946; doi:10.18632/oncotarget.21227)
Supplement: Supplementary file 1 [file oncotarget-08-91558-s001.pdf]

Integrative analysis to identify oncogenic gene expression changes associated with copy number variations of enhancer in ovarian cancer

SUPPLEMENTARY MATERIALS

Supplementary Table 1: The target genes of enhancers from ovary tissue in Human

See Supplementary File1

Supplementary Table 2: The copy number variations of all the ovary-specific enhancers in TCGA ovarian cancer cohort

See Supplementary File 2

Supplementary Table 3: The regulatory pairs between amplified enhancers and the targeting genes with concordant up-regulation in multiple TCGA ovarian cancer samples

See Supplementary File 3

Supplementary Table 4: The functional analysis results of 64 genes from high confidence enhancer regulation with evidence from 15 or more ovarian cancer samples

See Supplementary File 4

Supplementary Table 5: The identified regulations on the shared targeting genes by super-enhancers (multiple proximal enhancers)

See Supplementary File 5

Supplementary Table 6: The functional enrichment analysis of 210 genes regulated by super-enhancers

See Supplementary File 6

Supplementary Table 7: The prognostic Z-scores of 210 genes regulated by super-enhancers across 23 cancer types

See Supplementary File 7
